# Nafithromycin in CABP: promise and precaution—authors’ reply

**DOI:** 10.1016/j.lansea.2025.100696

**Published:** 2025-11-13

**Authors:** Himanshu Pophale, Monica Gupta, Hariharan Periasamy, Balaji Veeraraghavan, Sachin Bhagwat

**Affiliations:** a Jeevandhara Multispecialty Hospital, Pune, Maharashtra, India; b Samvedna Hospital, Varanasi, Uttar Pradesh, India; c Discovery Research, Wockhardt, Aurangabad (Chhatrapati Sambhajinagar), Maharashtra, India; d Department of Clinical Microbiology, Christian Medical College, Vellore, Tamil Nadu, India

We thank Gupta et al. for their observations,^1^ however their comments suggest a certain unfamiliarity with the regulatory and scientific principles that underpin the design and approval of new antibacterial therapies such as those for community-acquired bacterial pneumonia (CABP), a condition largely treated empirically in out-patient settings. Unlike academic or investigator-initiated studies, pivotal trials are undertaken in consultation with regulators, and in this instance, the 12.5% non-inferiority margin and the choice of moxifloxacin as comparator were fully aligned with regulatory requirements.^2^ Regulators require therapeutic equipoise, using a standard-of-care comparator with established efficacy and safety in the given indication demonstrated in prior RCTs. Accordingly, nafithromycin's global Phase 2 and India Phase 3 studies used moxifloxacin, consistent with CABP trials of lefamulin, omadacycline, and delafloxacin.^3^, ^4^, ^5^ While one may wish to consider alternative treatments such as amoxicillin combined with azithromycin or doxycycline as recommended in treatment guidelines, these regimens lack robust RCTs establishing a reliable magnitude of treatment effect and are therefore unsuitable as comparators in non-inferiority designs. In contrast, respiratory fluoroquinolones remain the most reliable monotherapy option for CABP, recommended for comorbid patients and in regions with high macrolide resistance, and their efficacy is supported by multiple recent pivotal RCTs. In this context, our trial is significant as it represents the first head-to-head comparison of a macrolide-class antibiotic with a respiratory fluoroquinolone. Further, primary endpoint based on early clinical response at the 3–5-day window, has been proven to capture the *clearest distinction* between effective and ineffective CABP therapy, also aligns with the clinical as well as regulatory requirements.^2^

In this pivotal study, a substantial proportion of real-world patients with PORT III and IV, severe yet treatable with oral antibiotics, were enrolled.^6^ Although regulatory guidance for outpatient therapies recommends inclusion of patients with only PORT class II or III, this study enrolled PORT IV patients as well, resulting in PORT III and IV categories collectively representing about 40% of the study population.^6^ The Authors appear to have overlooked that treatment differences in vulnerable patients-PORT III/IV or ≥65 years, clearly favored nafithromycin. Moreover, subjects had to meet rigorous disease diagnosis criteria, which included radiographic evidence and cardinal signs and symptoms of CABP, to be eligible for study enrollment,^6^ refuting the author's assertion of an inflated event rate.

While an ideal wish could be to confirm pathogens in most participants, pathogen isolation rates in real-world CABP trials reflect clinical practice where pathogen isolation rate is low and therapy is selected empirically. For instance, a recent Indian surveillance found only approx. 350 *Streptococcus pneumoniae* among 100,000 isolates^7^ and likewise global SENTRY surveillance has one of the lowest representation of this pathogen.^8^ This also reflects in global CABP trials such as, with lefamulin where *S. pneumoniae* isolation from the sputum culture was <5% of randomized patients.^9^ Nevertheless, despite the inherent limitation of low pathogen isolation rates from sputum samples, in our study, five patients with macrolide-resistant *S. pneumoniae* at baseline responded favorably to nafithromycin therapy. To draw a parallel, in the pivotal Phase 3 ceftazidime/avibactam trials for cUTI and cIAI, extremely small number of patients with carbapenem-resistant infections were enrolled due to the constraints of trial design.^10^^,^^11^ Nevertheless, following regulatory approval, ceftazidime/avibactam has been primarily used for the treatment of carbapenem-resistant infections, underscoring that limited trial representation does not preclude real-world utility against these highly resistant pathogens.

Thus, the choice of antibiotic in clinical practice is an '*art*' that integrates evidence from clinical trials, microbiological data, PK/PD features (demonstration of efficacy in translational animal models), and epidemiological trends.^12^ Considering the ∼75% macrolide non-susceptibility among *S. pneumoniae* isolates in India,^7^ nafithromycin's potent activity against macrolide-resistant *S. pneumoniae* (MIC_90_ 0.06–0.12 mg/L), its several-fold higher pulmonary exposure compared to azithromycin, and its demonstrated Phase 3 efficacy and safety^13^ collectively underscore its therapeutic value in the management of CABP including those caused by macrolide-resistant pneumococci.

## Contributors

Conceptualization: BV; Writing original draft: BV and SB; Writing review and editing: HP (Himanshu Pophale), MG, HP (Hariharan Periasamy), BV and SB.

## Declaration of interests

HP (Himanshu Pophale) and MG received the investigator grant from Wockhardt for the study conduct. HP (Hariharan Periasamy) and SB are employees of Wockhardt, India. SB owns stock or stock options in Wockhardt. BV has none to declare.
